# Exploring the potential of German claims data to identify incident lung cancer patients

**DOI:** 10.1186/s12890-025-03740-8

**Published:** 2025-06-26

**Authors:** Josephine Kanbach, Nikolaj Rischke, Sabine Luttmann, Ulrike Haug

**Affiliations:** 1 Department of Clinical Epidemiology, Leibniz Institute for Prevention Research and Epidemiology– BIPS, Achterstr. 30, 28359 Bremen, Germany; 2 Faculty of Human and Health Sciences, University of Bremen, Bremen, Germany

**Keywords:** Lung cancer, Claims data, Cancer registry data, Incidence, Survival

## Abstract

**Background:**

Real-world healthcare databases offer great potential for cancer research, but the valid identification of cancer patients is crucial for the suitability of a database in this regard. We aimed to assess the plausibility of an algorithm to identify incident lung cancer (LC) patients in German claims data.

**Methods:**

Using the German Pharmacoepidemiological Research Database (GePaRD; claims data from ∼ 20% of the German population) we applied a previously developed algorithm which identifies incident LC patients and classifies them into advanced and non-advanced. We calculated age-standardized incidence rates (ASIRs) per 100,000 for the years 2013–2018. Further, we assessed the ASIRs stratified by the deprivation index of the district of residence and determined age-standardized five-year absolute and relative survival. We stratified all analyses by sex.

**Results:**

Overall, we identified ∼ 9,500 − 10,500 incident LC patients per year. In 2018, (*N* = 10,625, mean age: 69.2 years in men) the proportion classified as advanced at diagnosis was 71.4%; the ASIRs of LC were 45 per 100,000 in men (9% lower than in 2013) and 27 per 100,000 persons in women (similar to 2013). ASIRs were lowest in persons living in areas with a low deprivation index. Age-standardized five-year absolute and relative survival rates, respectively, were 31% and 34% in women and 27% and 31% in men.

**Conclusions:**

The algorithm we applied to identify incident LC patients in German claims data yielded plausible results, supporting its validity.

**Trial registration:**

Not applicable.

**Supplementary Information:**

The online version contains supplementary material available at 10.1186/s12890-025-03740-8.

## Background

Cancer registries in Germany provide valuable data to describe the incidence, mortality and survival rate of cancer and to investigate differences by age, sex and region of residence. Information on tumor characteristics such as tumor stage is also available, although not always complete. Since 2013, clinical cancer registration has been established in Germany, collecting information on the treatment and course of cancer. Once this data is complete, it is intended to facilitate, among others, description of treatment patterns and assessment of the quality of care of cancer patients [1, 2].

However, there are also research questions requiring, for instance, information on comorbidities or other data not collected by cancer registries but available in different data sources such as health claims data. An important prerequisite for many of these research questions is the valid identification and classification of incident cancer diagnoses. For the use of claims data, this requires the development of sophisticated algorithms which also take into account potential miscoding. For colorectal cancer, we have shown– based on a comparison with cancer registry data– that the overall incidence as well as the distribution of tumor characteristics estimated based on such algorithms are very plausible [3, 4]. Regarding lung cancer (LC), German claims data have already been used for various research questions [5–8] but there was no study that systematically assessed the potential to identify incident LC diagnoses.

We therefore aimed to apply an algorithm for the identification of incident LC patients to a large German claims database and to indirectly validate it by assessing plausibility (1) of the LC incidence estimated based on this algorithm and (2) of the characteristics of identified LC patients regarding the age distribution at diagnosis, the proportion with advanced stage at diagnosis and survival.

## Methods

In the following sections, we describe the algorithm used to identify incident LC patients in a German claims database as well as the methods we used for its indirect validation, i.e. the assessment of its plausibility [9], which is to be distinguished from direct validation against a gold standard, which was not possible here (see discussion). To assess plausibility, we determined the LC incidence estimated based on the algorithm in men and women (overall and stratified by the deprivation index of the district of residence) as well as characteristics of identified LC patients (distribution of age, stage and survival stratified by sex). We conducted comparative analyses based on cancer registry data. Regarding the algorithm to identify incident LC cases, it is important to note that it was developed prioritizing a high specificity over a high sensitivity. This prioritization was guided by potential future applications in epidemiology. Methodologically, it can be shown that when outcome specificity is close to perfect and sensitivity is nondifferentially misclassified with respect to the exposure, the relative risk will be rather unbiased, i.e. optimizing specificity is important when investigating causal effects [10]. Identifying incident LC cases with high specificity means that one can be relatively certain that it is indeed an incident LC case. A high sensitivity, on the other hand, would mean that relatively few incident LC cases are overlooked, but this is usually accompanied by a reduction in specificity.

### Data sources

We used the German Pharmacoepidemiological Research Database (GePaRD) containing health claims data from four statutory health insurance (SHI) providers in Germany. GePaRD covers about 20% of the German population and all geographical regions of Germany are represented. Furthermore, data from another SHI provider (AOK Nordwest) were used, increasing the coverage particularly for the northwestern region of Germany. In addition to demographic data, German health claims data contain information on drug dispensations as well as outpatient (i.e. from general practitioners and specialists) and inpatient services and diagnoses. The diagnosis codes are recorded according to the International Statistical Classification of Diseases and Related Health Problems, 10th revision, German Modification (ICD-10 GM). Outpatient diagnosis codes are labeled as “confirmed”, “suspected”, “excluded” or “status post”.

Cancer registry data were provided by the German Centre for Cancer Registry Data (Zentrum für Krebsregisterdaten, ZfKD), which collects data from population-based cancer registries on the state level. We only included data from federal state cancer registries showing an estimated level of completeness of at least 90–95% with respect to the incidence of LC between 2013 and 2018. This applied to the following eight federal state cancer registries covering about 56,381,953 inhabitants of Germany (∼ 68% of the general population): Schleswig-Holstein, Hamburg, Lower Saxony, Bremen, North Rhine-Westphalia, Baden-Wurttemberg, Bavaria and Saarland. For the survival analyses, we further restricted the cancer registries to those with an overall proportion of LC-specific death certificate only cases below 15% between 2013 and 2018, a cutoff typically used for the analysis of ZfKD data, which applied to five out of these eight cancer registries [11].

### Identification and classification of incident lung cancer patients in claims data

To identify incident LC patients in claims data we used an algorithm which we developed based on case reviews, i.e. a comprehensive review of all information available in GePaRD (codes for any diagnoses, cancer-related treatment and diagnostic procedures in their chronological order) in randomly selected patients with at least one code (any type) for LC. Among others, these case reviews suggested that the vast majority of patients with only outpatient diagnosis codes for LC were unlikely to be true incident LC patients, i.e. including such patients would have substantially decreased specificity of the algorithm, which we wanted to avoid for reasons explained above. Accordingly, in a first step, the algorithm selects all patients with at least one inpatient discharge diagnosis code of LC (ICD-10 GM: C33-C34) and a continuous health insurance period of at least three years before the first LC diagnosis code (inpatient or outpatient) was recorded. Continuous health insurance means that patients had to be continuously insured with one of the included SHI providers, whereby a gap of 30 days is allowed. A pre-observation period is required to distinguish incident from prevalent cancers in claims data [12]. Patients with at least one “status post” diagnosis in the three-year pre-observation period are excluded. In the next step, patients are excluded if an inpatient discharge diagnosis of another cancer that is likely to metastasize to the lung was recorded before or in the quarter of the first inpatient LC diagnosis.

For included LC patients, the date of the first LC diagnosis code (inpatient or outpatient diagnosis labeled as “confirmed”) is assigned as date of diagnosis. Information on the UICC stage is not available in claims data but, as previously described for colorectal cancer [3], a rough stage at diagnosis is assigned (advanced vs. non-advanced). For this stage classification, codes for affected lymph nodes, distant metastases and other cancers likely indicating metastases rather than second primary tumors were considered if they were coded in the quarter of the LC diagnosis or the following quarter. In addition, patients with a LC diagnosis indicating a locally advanced stage (ICD-10 GM: C34.8) are assigned to the group “advanced stage at diagnosis” [5]. The category “missing” does not exist in claims data given that patients without codes indicating advanced stage are classified as non-advanced. The list of codes used for the analyses is available upon request from the corresponding author.

### Analysis of claims data

Based on the algorithm described above, we identified incident LC patients diagnosed between 2013 and 2018. We excluded those with inconsistent or missing information on sex or age as well as those not living in Germany. To determine year-wise incidence, we divided the number of included LC patients per year by the number of all persons insured in the respective year. For the latter, we applied the same exclusion criteria as for the numerator, i.e. inconsistent or missing information on sex or age, residence outside Germany and less than three years of continuous health insurance before the respective year. Stratified by sex, we calculated crude and age-standardized incidence rates (ASIRs) of LC per 100,000 for each year, using the old European Standard Population as reference.

Furthermore, we determined and compared ASIRs for persons living in a district with a high vs. a low deprivation index according to the German index of socioeconomic deprivation (GISD) [13]. In a sensitivity analysis, we determined ASIRs using a weighting factor to roughly account for the fact that the proportion of persons insured by the SHI provider “Allgemeine Ortskrankenkasse” (AOK), who tend to have a lower socioeconomic status, is lower in our study population as compared to the whole of Germany [14].

We calculated age-standardized five-year absolute and relative survival rates for the period 2013 to 2018 using period analysis [15] (data from 2008 to 2018 were used for the calculations). Relative survival rates were computed as a ratio of the observed survival of LC patients divided by the expected survival of the underlying population. Expected survival was calculated using national life tables stratified by age, sex and calendar year in line with the Ederer II method [16]. For age-standardization we used weights defined by the International Cancer Survival Standard [17].

### Analysis of cancer registry data

Analogously to the claims data analyses, we characterized the patients diagnosed between 2013 and 2018 regarding age and stage at diagnosis (advanced vs. non-advanced) and calculated ASIRs for each year between 2013 and 2018. LC cases with metastases, involved lymph nodes, T-stage 4 and cases with C34.8 were defined as advanced LC. The remaining were classified as non-advanced or as missing if there was no information on stage. As denominator, we used the number of inhabitants of the federal states included in the analysis [18]. We also determined age-standardized five-year absolute and relative survival rates for the period 2013 to 2018 analogously to the claims data analysis.

We conducted all analyses using SAS Software Version 9.4 [19]. For the survival estimates, we used SAS macros to perform period analysis and age-standardization [20, 21].

## Results

### Analysis of claims data

Overall, we identified 61,808 LC patients based on the algorithm (Additional file 1). The characteristics of these patients are shown in Table 1, exemplified for LC patients diagnosed in 2013 (*N* = 9,488) and 2018 (*N* = 10,625). In both years, the mean age at diagnosis was ∼ 69 years in men and 68–69 years in women. About 70% of LC patients were classified as advanced. About 17–18% of LC patients lived in districts with a low deprivation index and 15–17% lived in districts with a high deprivation index.

**Table 1:** Characterization of incident lung cancer patients identified in the claims database

	2013	2018
Overall, N	9488	10,625
Men, N (%)	5649 (59.5)	6056 (57.0)
Women, N (%)	3839 (40.5)	4569 (43.0)
Mean age at diagnosis (years)		
Men	68.8	69.2
Women	67.9	68.6
Stage at diagnosis^a^		
Non-advanced, N (%)	2934 (30.9)	3043 (28.6)
Advanced, N (%)	6554 (69.1)	7582 (71.4)
GISD^b^		
Low deprivation, N (%)	1626 (17.1)	1867 (17.6)
Medium deprivation, N (%)	6276 (66.2)	7151 (67.3)
High deprivation, N (%)	1585 (16.7)	1607 (15.1)

^a^ The category “missing” does not exist in claims data. If there were no codes indicating advanced disease (see methods section) the patient was classified as non-advanced

^b^ German Index of Socioeconomic Deprivation. Quintiles classified into three categories: Low deprivation (1st quintile), medium deprivation (2nd– 4th quintile) and high deprivation (5th quintile). The GISD was assigned based on the district of residence

**Fig. 1:**
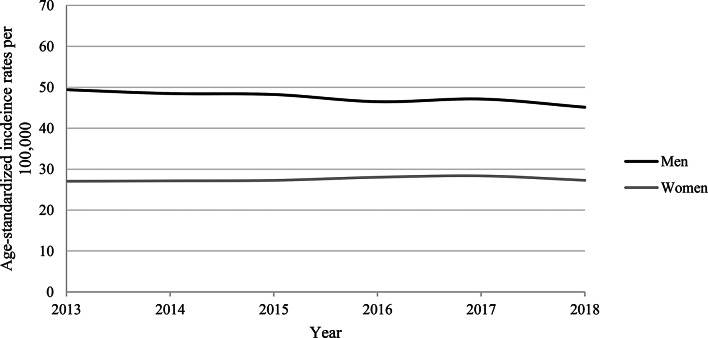
Age-standardized incidence rates per 100,000 in claims data, stratified by sex

**Fig. 2:**
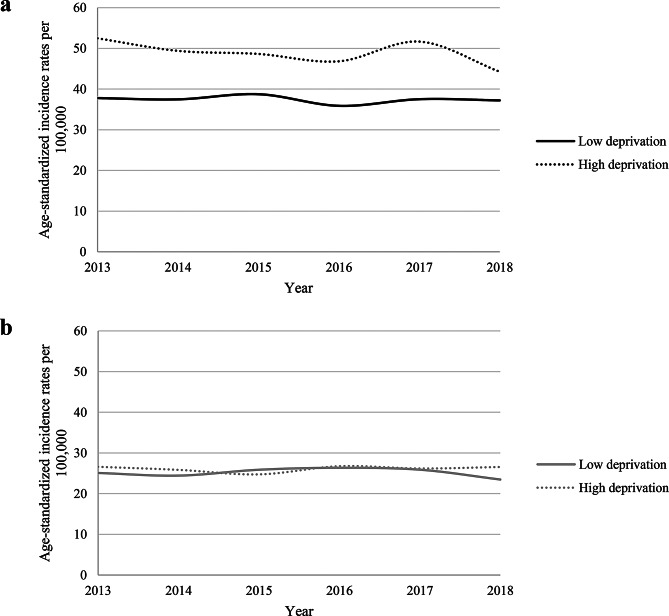
**(a)** Age-standardized incidence rates (ASIRs) per 100,000 in claims data among men living in a district with a low vs. a high deprivation index. **(b)** Age-standardized incidence rates (ASIRs) per 100,000 in claims data among women living in a district with a low vs. a high deprivation index

Figure 1 shows the ASIRs stratified by sex in claims data. In men, the ASIRs decreased from 49.4 per 100,000 in 2013 to 45.1 per 100,000 in 2018. In women, the ASIRs remained relatively stable between 2013 and 2018 at ∼ 27 per 100,000 persons. As shown in Fig. 2a, the ASIRs among men were ∼ 11% points higher in districts with a high deprivation index as compared to districts with a low deprivation index. Among women (Fig. 2b), no such differences according to deprivation index were observed.

**Fig. 3:**
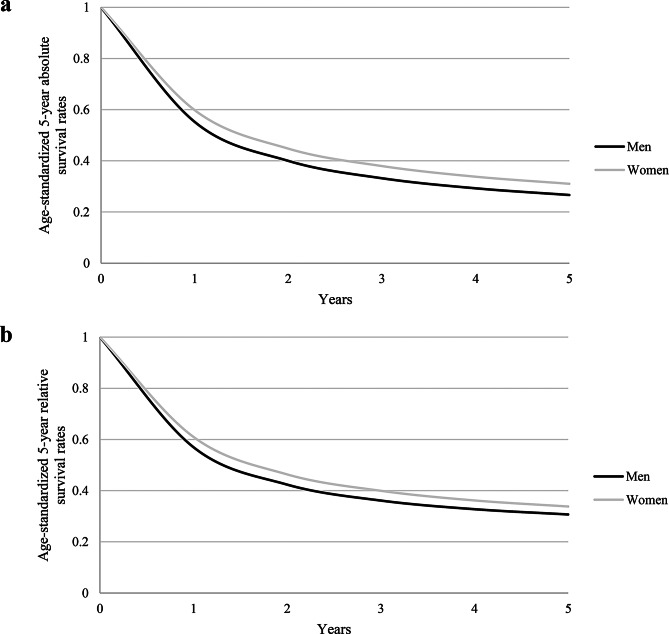
**(a)** Age-standardized absolute survival rates in claims data, stratified by sex. **(b)** Age-standardized relative survival rates in claims data, stratified by sex

As shown in Fig. 3a, the age-standardized one-year absolute survival was 60.0% in women and 55.0% in men. After five years, it was 31.0% in women and 26.7% in men. Age-standardized five-year relative survival rates were 30.7% in men and 33.8% in women (Fig. 3b).

### Analysis of cancer registry data

**Fig. 4:**
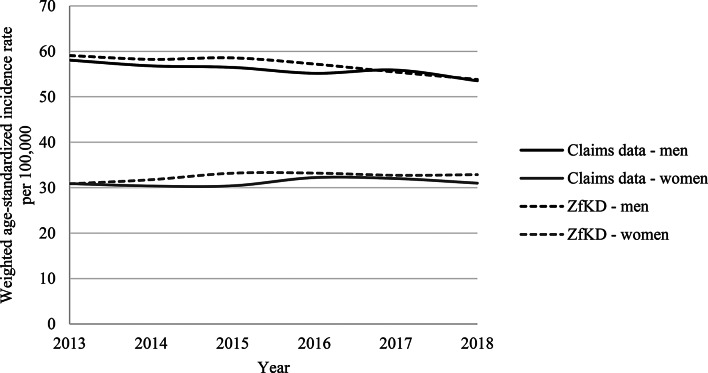
Age-standardized incidence rates (ASIR) per 100,000 in claims data, (here roughly weighted to take into account differences between health insurance providers) and in cancer registry data (ZfKD), stratified by sex

As shown in Additional file 2, the mean age at diagnosis in the years 2013 and 2018 was 69–70 years in men and 68–69 years in women. The proportion of LC patients with an advanced stage at diagnosis among those with available information on stage was ∼ 78% in 2013 and 2018. The information on stage was missing in 35-36% for both years. As shown in Fig. 4, the ASIRs decreased in men from 59.1 per 100,000 in 2013 to 53.8 per 100,000 persons in 2018. In women, the ASIR was 30.9 per 100,000 in 2013 and 32.9 per 100,000 in 2018. As also shown in Fig. 4, the ASIRs determined based on claims data and weighted to adapt the proportion of patients insured by the AOK to the whole of Germany were similar or approached the ASIRs determined based on cancer registry data.

As shown in Additional file 3, the age-standardized one-year absolute survival was 53.6% in women and 46.3% in men. The five-year absolute survival was 23.5% in women and 17.5% in men. Age-standardized five-year relative survival rate was 20.2% in men and 25.5% in women (Additional file 4).

## Discussion

Our study, which is the first to systematically assess feasibility of identifying incident LC patients in German claims data, showed that applying a strict algorithm to define incident LC patients yields plausible results, i.e. this indirect validation approach supports the validity of the case definition. Plausibility was supported by LC incidence rates in men and women overall and stratified by the deprivation index of their district of residence, by a detailed characterization of incident LC patients regarding the distribution of age, (rough) stage at diagnosis and survival as well as by comparative analyses using cancer registry data.

Unlike previous studies using German claims data [5, 6], the algorithm we used also considered potential miscoding of LC among patients with other cancers that metastasize to the lung if they were coded in the quarter of the LC diagnosis or the following quarter. The necessity of this component of the algorithm became evident when reviewing patient profiles. It was observed, for example, that in hospitalized breast cancer patients, codes for both lung metastases and LC were recorded. If this miscoding was not taken into account, the incidence of LC would be overestimated. In total, 9,559 out of 71,464 patients (13.4%) in our study population were excluded on the basis of this criterion, so it is rather relevant to be considered if the aim is to identify LC patients in claims data with a high specificity.

As expected, the incidence of LC observed in our study was lower than the incidence of LC determined based on cancer registry data. In Germany, there are different statutory health insurance providers whose insured populations differ in terms of socioeconomic factors and therefore presumably also in terms of smoking prevalence [22, 23]. In our sensitivity analysis, where we weighted the proportion of patients insured by the AOK, who tend to have a lower socioeconomic status, to make our sample more representative of the whole population, the ASIRs determined based on claims data were rather comparable to those determined based on cancer registry data. This was reassuring given that the algorithm we used prioritizes a high specificity over a high sensitivity, i.e. priority is given to being sure that identified LC patients indeed have LC rather than to making sure that no LC patient is missed. Based on these comparative analyses, it does not seem that a high proportion of LC patients is missed with the algorithm, although those that are missed might be a selective group of patients, such as older, late-stage LC patients who are not hospitalized but receive only outpatient palliative care.

The aforementioned aspect, i.e. the differences in the underlying populations regarding socioeconomic status and smoking prevalence, might also explain why there was no full agreement with respect to survival between both data sources. Also, among those developing LC, the smoking history (proportion of non-smokers, intensity of smoking prior to and after diagnosis) is known to impact on survival [24–27], i.e. it seems plausible that the survival rates observed in our claims data analysis were somewhat higher than those determined based on cancer registry data. In addition, the difference in the proportion of LC diagnosed at an advanced stage (claims data: ∼70%, cancer registries: ∼78%) might have contributed to the difference in survival. With its focus on a high specificity, the algorithm might have missed some LC cases receiving only palliative care in the outpatient setting. However, given that information on stage was missing in almost one third of patients in the cancer registry data, it is not clear whether there is a true difference in the distribution of stage, so this remains speculative. Regarding the question whether survival by stage in claims data is plausible, we showed in a previous study focussing on targeted therapies that, as expected, survival was markedly lower for advanced as compared to early stages [28].

In GePaRD, the LC incidence rate was higher among men living in districts with a higher as compared to a lower deprivation index, while no such a difference was observed among women. Although this seems surprising, it is consistent with the literature. Hoebel et al. systematically assessed overall and site-specific cancer incidence according to the deprivation index based on German cancer registry data. Similarly to the patterns in GePaRD, they found socioeconomic inequalities in cancer incidence in men but not in women [29].

Our study has limitations that should be mentioned. First, to investigate socioeconomic differences, we could only use the deprivation index of the district of residence as an indicator of the socioeconomic status. A categorization of the deprivation index on a smaller area level would certainly be more informative. For example, all persons living in the federal state of Bremen (one district) were assigned to the same deprivation index, even though there are marked differences regarding educational and unemployment status between the neighborhoods [30]. Nevertheless, even based on this rough categorization we observed socioeconomic differences in incidence rates as expected. As an alternative to estimating the deprivation index based on the place of residence, GePaRD also contains information on the educational level, but for persons older than 65 years this information is often missing [31], so we did not use it in this study. Second, as we argued before, the differences in incidence rates between cancer registries and GePaRD are most likely explained by differences in the smoking prevalence. To demonstrate this, information on the smoking status would have been useful but this was not available in either data source. Third, we only included data from federal state cancer registries showing an estimated level of completeness of at least 90–95% with respect to the incidence of LC between 2013 and 2018. As a consequence, federal state cancer registries from the eastern part of Germany were underrepresented in our study. There are some geographical differences in LC incidence in Germany. For example, age-standardized LC incidence in 2015 in men was about 3% higher and in women, it was about 10% lower in the former East as compared to former West Germany [32]. On the other hand, also the GePaRD database has a higher population coverage in the western as compared to the eastern part of Germany. Combined with the fact that the geographical differences in LC incidence are not large, we believe that our comparisons have hardly been affected. Lastly, we used the approach of indirect validation including a comparison of claims data and cancer registry data on an aggregated level. A direct comparison of claims data and cancer registry data based on a linked dataset was not possible in the context of our study. There are some studies from other countries conducted several years ago that used linkage with cancer registry data to validate algorithms for the identification of (lung) cancer diagnoses in healthcare data. Although it has to be kept in mind that comparability is limited as coding practices vary between healthcare systems, these studies showed a high accuracy for the identification of LC cases [33, 34].

## Conclusions

In conclusion, our approach based on indirect validation supports the possibility to identify incident LC patients in German claims data. Identifying LC patients in claims data opens up the possibility of addressing questions on LC that require information on comorbidity or comedication, for example. This means that health insurance data can be a valuable addition to cancer registry data, which in turn is better suited for questions that require more detailed information on the tumor (e.g. histology). A linked data set combining the advantages of both data sources would also be a promising option for future research on LC.

## Electronic supplementary material

Below is the link to the electronic supplementary material.

Supplementary Material 1: Additional file 1: Flow chart illustrating the stepwise identification of incident lung cancer patients in claims data. (DOCX 38 kb)

Supplementary Material 2: Additional file 2: Characterization of incident lung cancer patients identified in cancer registry data (exemplified for the years 2013 and 2018). (DOCX 15 kb)

Supplementary Material 3: Additional file 3: Age-standardized absolute survival rates in claims data (GePaRD) and in cancer registry data (ZfKD), stratified by sex. (DOCX 16 kb)

Supplementary Material 4: Additional file 4: Age-standardized relative survival rates of lung cancer in claims data (GePaRD) and cancer registry data (ZfKD) stratified by sex. (DOCX 16 kb)

## Data Availability

As we are not the owners of the data we are not legally entitled to grant access to the data of the German Pharmacoepidemiological Research Database. In accordance with German data protection regulations, access to the data is granted only to employees of the Leibniz Institute for Prevention Research and Epidemiology– BIPS on the BIPS premises and in the context of approved research projects. Third parties may only access the data in cooperation with BIPS and after signing an agreement for guest researchers at BIPS. In order to obtain access to the raw data, please contact the corresponding author, namely Prof. Ulrike Haug (haug@leibniz-bips.de).

## Abbreviations

AOK: Allgemeine Ortskrankenkasse
ASIRs: Age-standardized incidence rates
GePaRD: German Pharmacoepidemiological Research Database
GISD: German Index of Socioeconomic Deprivation
ICD-10 GM: International Statistical Classification of Diseases and Related Health Problems, 10th revision, German Modification
LC: Lung cancer
SHI: Statutory health insurance
UICC: Union internationale contre le cancer
ZfKD: German Centre for Cancer Registry Data
