# Supplementary material for: Exploring the potential of German claims data to identify incident lung cancer patients
Source: BMC Pulm Med. 2025 Jun 26;25:289. doi: 10.1186/s12890-025-03740-8 (PMC12203718; doi:10.1186/s12890-025-03740-8)
Supplement: Supplementary file 1 — Supplementary Material 1: Additional file 1: Flow chart illustrating the stepwise identification of incident lung cancer patients in claims data. (DOCX 38 kb) [file 12890_2025_3740_MOESM1_ESM.docx]

**Additional file 1:** Flow chart illustrating the stepwise identification of incident lung cancer patients in claims data.

All patients with an in- or outpatient diagnosis code of lung cancer (C33-C34) between 2013 and 2018
N = 102585

Exclusion of patients with a pre-observation period less than three years
N = 7119

N = 95466

^[[1]](#footnote-1)^

Exclusion of patients with outpatient diagnoses only^a^

N = 23737

N = 71729

Exclusion of LC patients with at least one “status post” diagnosis in the pre-observation period of three years
N = 265

N = 71464

Exclusion of patients when a cancer diagnosis likely to metastasize to the lung is coded in the quarter of the LC diagnosis or the following quarter
N = 9559

N = 61905

Exclusion of LC patients with missing or inconsistent information on sex or birth year and LC patients not living in Germany
N = 97

N = 61808

1. a The patients’ profile reviewing considering also treatment and procedure codes showed that such patients were clearly not incident cases (possibly prevalent cases with an inpatient diagnosis that is not visible in the data due to left truncation of the time axis) or were not LC cases at all (miscoding). [↑](#footnote-ref-1)
