# Supplementary material for: Exploring the potential of German claims data to identify incident lung cancer patients
Source: BMC Pulm Med. 2025 Jun 26;25:289. doi: 10.1186/s12890-025-03740-8 (PMC12203718; doi:10.1186/s12890-025-03740-8)
Supplement: Supplementary file 2 — Supplementary Material 2: Additional file 2: Characterization of incident lung cancer patients identified in cancer registry data (exemplified for the years 2013 and 2018). (DOCX 15 kb) [file 12890_2025_3740_MOESM2_ESM.docx]

Additional file 2: Characterization of incident lung cancer patients identified in cancer registry data (exemplified for the years 2013 and 2018).

|  |  | 2013 |  | 2018 |
| --- | --- | --- | --- | --- |
| Overall, N |  | 36458 |  | 37896 |
| Men, N (%) |  | 23176 (63.6) |  | 22802 (60.2) |
| Women, N (%) |  | 13282 (36.4) |  | 15094 (39.8) |
| Mean age at diagnosis (years) |  |  |  |  |
| Men |  | 69.4 |  | 70.0 |
| Women |  | 68.2 |  | 69.0 |
| Stage at diagnosis^a,b^ |  |  |  |  |
| Non-advanced, N (%) |  | 5144 (22.0) |  | 5369 (22.0) |
| Advanced, N (%) |  | 18192 (78.0) |  | 19108 (78.1) |

^a^ Localized tumors (Tumor size < T4) were defined as non-advanced LC and cases with present metastases, involved lymph nodes as well as Tumor size T4 or an ICD-10 diagnostic code C34.8 were defined as advanced LC.

b Information about stage was missing in 13,122 (36.0%) LC cases in 2013 and 13,419 (35.4%) LC cases in 2018.
