# Supplementary material for: Exploring the potential of German claims data to identify incident lung cancer patients
Source: BMC Pulm Med. 2025 Jun 26;25:289. doi: 10.1186/s12890-025-03740-8 (PMC12203718; doi:10.1186/s12890-025-03740-8)
Supplement: Supplementary file 3 — Supplementary Material 3: Additional file 3: Age-standardized absolute survival rates in claims data (GePaRD) and in cancer registry data (ZfKD), stratified by sex. (DOCX 16 kb) [file 12890_2025_3740_MOESM3_ESM.docx]

Additional file 3: Age-standardized absolute survival rates in claims data (GePaRD) and in cancer registry data (ZfKD), stratified by sex.
